# Network-medicine framework for studying disease trajectories in U.S. veterans

**DOI:** 10.1038/s41598-022-15764-9

**Published:** 2022-07-14

**Authors:** Italo Faria do Valle, Brian Ferolito, Hanna Gerlovin, Lauren Costa, Serkalem Demissie, Franciel Linares, Jeremy Cohen, David R. Gagnon, J. Michael Gaziano, Edmon Begoli, Kelly Cho, Albert-László Barabási

**Affiliations:** 1 Center for Complex Network Research, Department of Physics, Northeastern University, Boston, USA; 2 Massachusetts Veterans Epidemiology and Research Information Center (MAVERIC), VA Boston Healthcare System, Boston, USA; 3 School of Public Health, Department of Biostatistics, Boston University, Boston, USA; 4 Oak Ridge National Laboratory, Oak Ridge, USA; 5 Department of Medicine, Brigham and Women’s Hospital and Harvard Medical School, Boston, USA; 6 Department of Medicine, Harvard Medical School, Boston, USA; 7 Brigham and Women’s Hospital, Harvard Medical School, VA Boston Healthcare System, 150 S. Huntington Avenue, Boston, 02130 USA

**Keywords:** Diseases, Computational biology and bioinformatics

## Abstract

A better understanding of the sequential and temporal aspects in which diseases occur in patient’s lives is essential for developing improved intervention strategies that reduce burden and increase the quality of health services. Here we present a network-based framework to study disease relationships using Electronic Health Records from > 9 million patients in the United States Veterans Health Administration (VHA) system. We create the Temporal Disease Network, which maps the sequential aspects of disease co-occurrence among patients and demonstrate that network properties reflect clinical aspects of the respective diseases. We use the Temporal Disease Network to identify disease groups that reflect patterns of disease co-occurrence and the flow of patients among diagnoses. Finally, we define a strategy for the identification of trajectories that lead from one disease to another. The framework presented here has the potential to offer new insights for disease treatment and prevention in large health care systems.

## Introduction

Diseases do not occur in isolation but usually co-occur with other disorders due to common genetic or environmental factors^1^. The prevalence of patients living with multiple conditions – referred to as comorbidity or multimorbidity—has been increasing^2^ and is reported to reduce life expectancy and to increase health-care costs^3^. Additionally, patients with multiple conditions are more frequent users of ambulatory and inpatient care, and experience reduced quality of life and clinical outcomes^4–7^. We must, therefore, develop improved intervention strategies that reduce the burden of comorbidity and increase the quality of health-care services. For this, we need a better understanding of the relationship among diseases together with the sequential and temporal aspects in which they emerge throughout a patient’s life.

The bulk of our current understanding on disease comorbidities and progression is derived from hypothesis-driven studies that focus on a specific disease and most prevalent comorbidities. In contrast, network medicine-based strategies offer tools to systematically explore the correlations across hundreds of diagnoses based on the analysis of Electronic Health Records (EHR). These methodologies have allowed the identification of disease comorbidities driven by demographic factors^8^, age^9^, gender^9–11^, genetics^12^, and environmental factors^13^ (Supplementary Table S1). Previous studies have also investigated disease progression in patients, allowing the discovery of trajectories related to chronic obstructive pulmonary disease, prostate cancer, and cerebrovascular disorders^14–19^ (Supplementary Table S1). Additionally, methodologies based on sequential pattern mining have also been used to identify temporal patterns of disease progression in EHR datasets, finding patterns related to the diagnosis of pediatric asthma, acute coronary syndrome, colorectal cancer and other conditions^20–23^. However, each study is heavily dependent on the characteristics of the underlying cohort, *i.e.* ancestry, age distribution, etc., which limits our ability to translate these findings to other populations. Therefore, it is necessary to revisit these methodologies in different contexts to find new patterns of disease comorbidity and progression, as well as to validate and confirm the findings from studies in other populations.

Here we analyzed the health records of the United States Veterans Health Administration (VHA), representing the largest single payer healthcare system in the USA. We relied on patient-specific information from approximately 9 out of 24 million veterans across 20 years of database records, mostly male patients (92%), from diverse ancestry backgrounds (e.g. Caucasians, Afro-Americans). Since the VHA system provides free healthcare for American veterans, most patients have all their medical care within this system, allowing us to compile longitudinal data for most individuals.

Our goal is to study disease progression in the VHA system to identify disease properties from the network that correlate with patient prognosis, describe co-occurrence patterns among diagnoses, and derive trajectories that explain the progression from one disease to another. We start by translating patients’ medical records into a network, where nodes are individual diagnosis and edges represent the number of patients that progress from one disease to another. We find disease properties in the network that correlate with patient survival and describe disease groups that tend to co-occur in patients. Finally, we define a method to identify disease trajectories between pairs of diseases that often co-occur in patients.

## Results

### Disease network

The largest single-payer health-care entity in the US, the VHA system, contains over 144 hospitals and 1,221 outpatient centers. Local hospital and clinic data, including inpatient, outpatient, laboratory values, and vital signs, are stored in a central VHA corporate data warehouse. Here, we analyzed outpatient visits recorded in the database (i.e., patients that visit the hospital but are not hospitalized) of male Veterans, comprising a cohort of 9,805,451 individuals, approximately 40% of all patients in the VHA database between 2002 and 2018. The inpatient records (i.e., hospitalized patients) were not considered in this study since they are related to the management of chronic and recurrent diseases, with particular properties, such as differences in diagnosis prevalence and correlations of specific diagnosis to either inpatient or outpatient records^24^.

**Figure 1:**
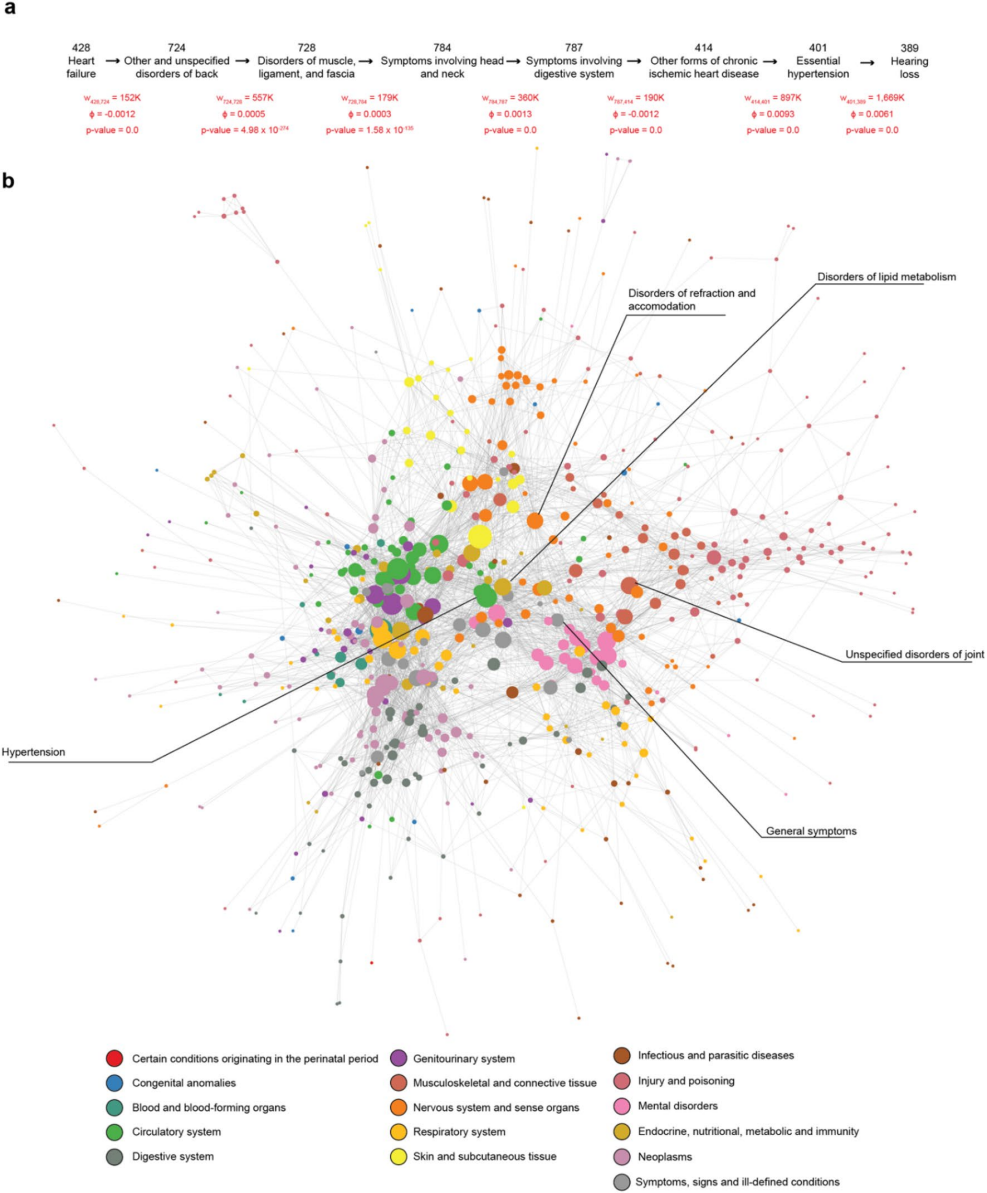
Temporal Disease Network. (**a**) Example of the disease records for a single patient and its representation into disease paths. The Disease Network connects diseases that occur consecutively in patients' records. Edge weights *w*_*ij*_, p-values, and $\phi$ values are shown for raw data and represent the number of patients, the correlation coefficient, and the significance, respectively, for each progression step. (**b**) Nodes represent diagnoses (ICD9 at the 3-digit level) and links represent the number of patients with disease A before diseases B. For visualization purposes, the edge directions were merged as single undirected edges, edges with $\phi$ < 0.001 were filtered, and disconnected nodes resulting from this filtering were omitted. The full network contains 718 nodes and 60,425 edges, while the visualization shows 638 nodes and 4,582 edges. The labels highlight diseases mentioned throughout the text and their corresponding nodes in the network.

Each record consists of the date of visit and one or multiple diagnosis, which are specified via standardized ICD-9-CM codes. ICD-9 codes contain up to 5 digits, the first three specifying the main disease category and the last two providing additional information about the disease. In total, the ICD-9-CM classification consists of 1,234 diagnoses at the 3-digit level and 17,561 diagnoses at 5 digits. In a tradeoff between power and specificity, we worked with ICD-9 at the third level. For a detailed list of currently used ICD9 codes see www.icd9data.com. We organized each patient’s medical history as a path: a list of ICD-9 diagnosis codes at the three-digit level ordered by the visit date of their first occurrence (Fig. 1a). If a patient had several diagnoses for the first time in the same visit date, all diagnoses were represented in the patient’s records. Using these individual paths, we built a directed network in which nodes are ICD-9 codes and the links represent the number of patients *w*_*ij*_ that have a diagnosis *i* followed by a diagnosis *j* (Fig. 1a). We performed filtering procedures to eliminate possible errors and biases in the data as well as based on the statistical significance of each link (see “Methods”), resulting in a network of 718 nodes and 60,425 edges (Fig. 1b). The resulting Temporal Disease Network (TDN), instead of encapsulating only undirected correlational evidence^8^ or enforcing a specific direction to edges^24^, contains both directions in which one disease might succeed or precede another one.

We start characterizing the TDN by evaluating a series of network measures. First, we evaluated the weighted degree (K_w_) of each node in the network, *i.e.* the sum of outgoing and incoming patients with a given disease in the network. Second, we evaluated diseases that receive high flow of patients by using a random walk-based measure, often used to evaluate the effects of network topology on patterns of flows through nodes, providing an intuitive interpretation of how real flows of patients take place in TDN^25^. We define flow as the expected density of random walkers on a node at stationarity, which can be measured by the global metric PageRank (PR). Finally, we evaluated diseases that intermediate connections among others by using the metric Betweenness Centrality (BC). Note that PR and BC represent global properties in TDN, where each disease is evaluated in relation to all others.

**Figure 2:**
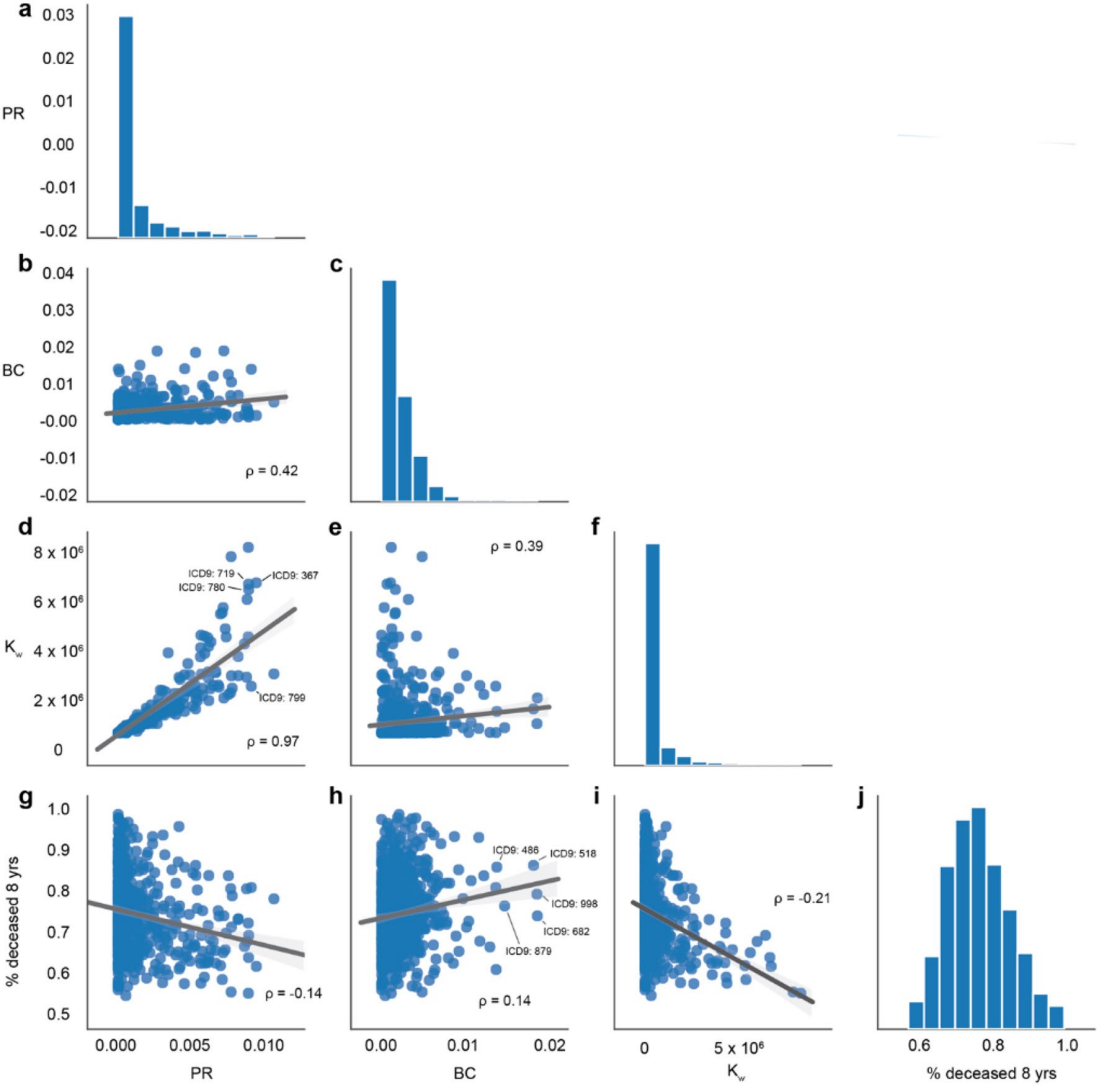
Network centrality and fatality. Comparison of network centrality and fatality values across nodes of the Disease Network. Inset numbers represent the Spearman correlation coefficient (all with p < 0.05).

We find that measures PR (Fig. 2a) and K_w_ (Fig. 2f) are highly correlated (Spearman *r* = 0.97) (Fig. 2d) with “disorders of refraction and accommodation” (ICD9: 367), “general symptoms” (ICD9: 780) and “other and unspecified disorders of joint” (ICD9: 719) ranking among the top 5 diseases by both measures. However, differences can be observed in the rankings provided by the different measures. For example, the diagnosis “other ill-defined and unknown causes of morbidity and mortality” (ICD9:799) is in the third position of the PR ranking, while it is in the 42nd position in the ranking provided by K_w_. We find that BC (Fig. 2c) shows low correlation with the other two measures (Spearman *r* = 0.42 and *r* = 0.39 in relation to PR and K_w_, respectively) (Fig. 2b,e). The top 5 diseases ranked by BC are “other cellulitis and abscess” (ICD9: 682), “other diseases of lung” (ICD9: 518), “pneumonia, organism unspecified” (ICD9: 486), “other complications of procedures, NEC” (ICD9: 998), and “open wound of other and unspecified sites, except limbs” (ICD9: 879).

To test whether the properties of the diseases in the network reflect true clinical aspects, we compared the centrality measure of each disease with a metric of fatality: the percentage of patients that die after 8 years of the first diagnosis for that disease (Fig. 2j). We observe that PR and K_w_ negatively correlate with fatality (Spearman *r* = −0.14 and *r* = −0.21, respectively) (Fig. 2g,i), suggesting that diagnoses highly ranked by these measures correspond to diagnoses that are common and generally observed in patients, while BC positively correlates with fatality (Spearman $r$ = 0.14) (Fig. 2h). Altogether these results demonstrate that the TDN extracted from EHR system of the VHA offers an accurate global picture of disease co-occurrence patterns.

### Communities

**Figure 3:**
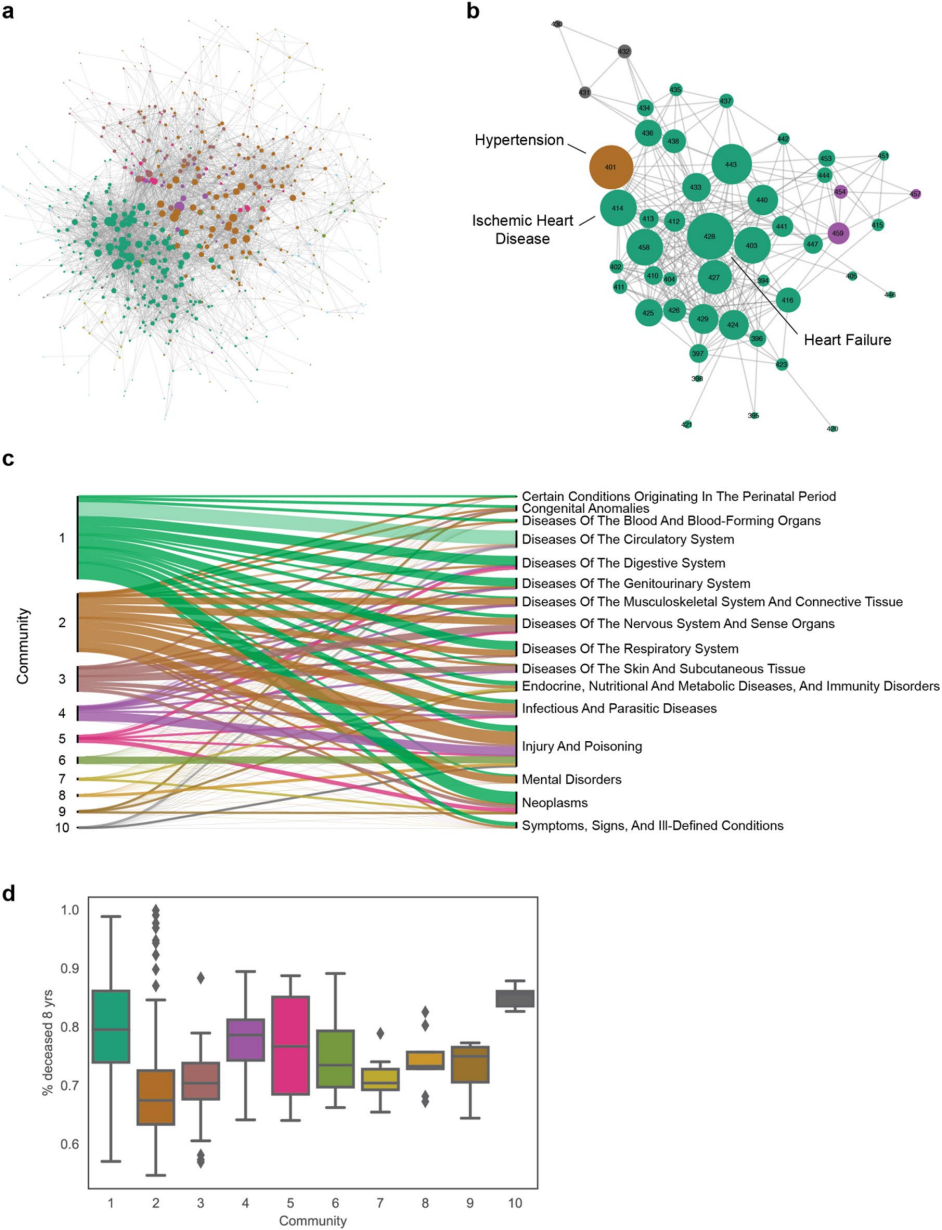
Communities. (**a**) Visualization of TDN with colors representing the different communities detected using InfoMap. (**b**) The community assignments for diagnoses in the ICD-9 chapter “Diseases of The Circulatory System”. (**c**) Alluvial diagrams representing the re-assignment of diseases from different ICD-9 chapters into the detected communities. (**d**) Distribution of % of deceased patients after 8 years of first diagnosis for diseases assigned in the different communities.

We next searched for disease groups, called communities, that tend to co-occur frequently among themselves, compared to diseases that are not members of the community^26^. ICD-9 codes are usually grouped in chapters, the highest-level categorization of diseases in the ICD-9 hierarchy. The diseases are grouped in categories defined by medical committees and represent the current state of art for clinical practice. A data driven approach to categorize diseases that captures the intricate disease co-occurrences might be better suited for clinical practice. To accomplish this, we applied the community detection algorithm InfoMap^27^, an information-theoretic method that uses random walks to evaluate the flow of information among the nodes of a network (see “Methods”). We identified a total of 29 communities and, after removing those with less than 5 diseases, we arrived at a final list of 10 communities (Fig. 3a). We labeled these communities TDN1 to TDN10, and the lower the index the higher the flow detected among nodes of that community, i.e., the higher the number of patients that go from one disease to another in that community. Some diseases from the different ICD-9 chapters were re-classified into different communities, such as the diseases in the chapter “Diseases of The Circulatory System”, which were assigned to five communities (TDN1-4 and TDN10) (Fig. 3b, c). The results indicate that the communities detect relationships among diseases that go beyond the ICD-9 chapter categorization. For example, all diseases in the community TDN7 are related to the thyroid organ and all diseases in TDN10 are related to cerebral hemorrhage, even though, in both cases, the diseases were divided in two ICD-9 chapters: Endocrine Nutritional and Metabolic Diseases and Neoplasms; and Diseases of Circulatory System and Injury and Poising; respectively. We evaluate the profile of the communities in terms of variety of ICD-9 codes represented in each community by defining the *H* score (see “Methods”), that ranges from 0, when all diseases are from the same chapter, to 1, when diseases are evenly distributed across chapters. Two communities resulted with $H$ scores of 0, the first containing 26 diagnoses related to bone fraction (TDN6), and the second containing 9 diagnoses related to burn (TDN8) (Table S1). Other communities with low $H$ scores represent diseases that are classified in different categories but are closely related to each other, such as the communities TDN7 (H = 0.19, 8 diagnoses), related to thyroid diseases, and TDN10 (H = 0.23, 5 diagnoses), related to cerebral hemorrhage.

These findings suggest that the network can reveal groups of diseases that are mechanistically or physiologically related, possibly suggesting new frameworks for disease classification. We compared the fatality among the different communities, finding that certain communities contain more severe diseases than the others (Fig. 3d). The communities with the highest severity were TDN10 and TDN1, with an average percentage of deceased patients after 8 years (after first disease diagnosis) of 85% and 81%, respectively, while the communities that had the lowest averages of deceased patients were TDN2 and TDN3 with 68% with 70%, respectively.

These results suggest a new approach to disease categorization. The ICD-9 framework divides diseases in chapters, such as Circulatory System and Respiratory System, for example. However, the assignment of diseases in such categories is often dependent on a canonical understanding of disease etiology that is based on the primary organ or system affected by the condition. A disease categorization based on the network communities relies on a data-driven framework, directly related to how diseases co-occur with each other in patient trajectories, and therefore more likely to highlight disease relationships of clinical relevance due to shared underlying molecular or environmental etiology.

### Disease trajectories

Next, we demonstrate that a network-based representation of medical records can help identify trajectories of diagnosis that link diseases with high comorbidity in the population. For example, diabetes is a well-known risk factor for cardiovascular disorders, such as stroke^28^. In practice, however, patients often show multiple and diverse diagnoses as they progress from diabetes to stroke. To describe the possible trajectories connecting two comorbid diseases, we first invert the weights of the links of the original network (i.e. ${w}_{ij}^{-1}$), so that the links with high comorbidity have smaller weights, hence have higher proximity. Next, we selected the path with the smallest weighted shortest path (i.e. $\sum {w}_{ij}$) as the most likely trajectory to connect the two diagnoses.

**Figure 4:**
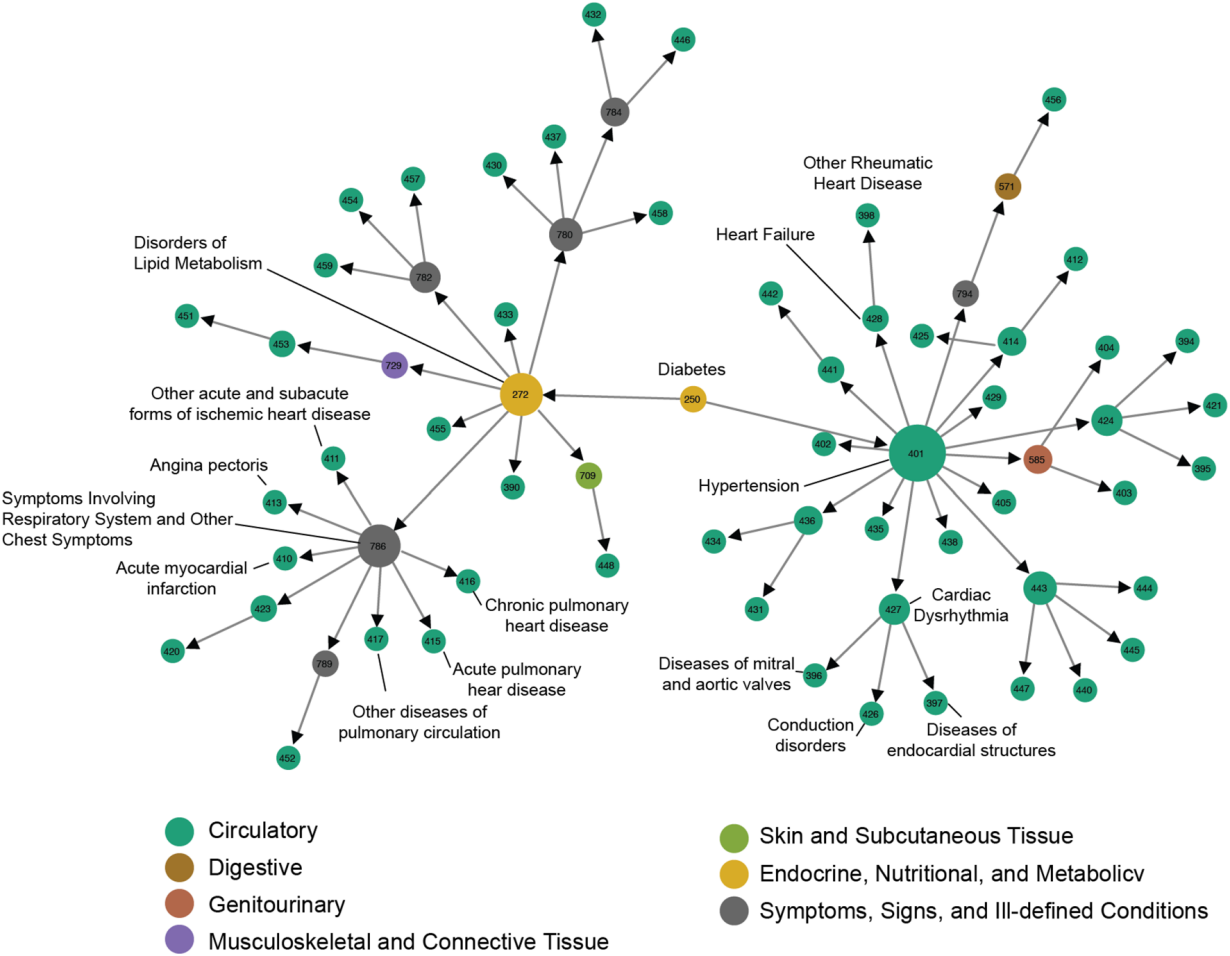
Disease Trajectories. Trajectories connecting diabetes mellitus to all diseases in the ICD-9 chapter “Diseases Of The Circulatory System”.

We demonstrate the trajectories obtained in TDN by aggregating the shortest paths connecting diabetes (ICD9:250) to all diseases of the circulatory system (Fig. 4). For example, the trajectories obtained from TDN contains 12 diagnoses not included in the circulatory system category. “Disorders of lipid metabolism” is one of the major intermediates of shortest paths connecting diabetes and CVs, which is in line with the clinical observation that insulin resistance leads to major vascular problems^29^, that, when combined with dysfunctional lipid metabolism, can lead to the formation of thrombus stroke^28^.

The trajectories highlight overall trends on how diagnoses are made in the VHA clinical practice. For example, the symptom “Cardiac dysrhythmia” precedes the more specific diagnoses that can cause it, such as “Diseases of endocardial structures”, “Conduction disorders” and “Diseases of mitral and aortic valves” (Fig. 4). The trajectories also show the role of more general and nonspecific diagnoses from the chapter “Symptoms, signs, and ill-defined conditions”. For example, all diagnoses related to pulmonary circulation (ICD9:415–417) and diagnoses related to acute ischemic heart disease (ICD9:410–411,413) tend to be preceded by the diagnosis “Symptoms involving respiratory system and other chest symptoms”.

These results suggest that the proposed framework to detect disease trajectories can reveal important patterns in disease progression. For example, previous studies report different patterns of comorbidities between ethnic and age groups^8,9^, suggesting that different disease trajectories might take place in individuals from these groups. Altogether, the framework presented in this study might reveal patterns of disease trajectories with potential to lead to better disease treatment and prevention.

## Discussion

Here we introduced a temporal network-based framework to analyze the electronic health records (EHRs) of over 9 million U.S. veterans. We first demonstrated that properties of the diseases in the network reflect fatality. Then, we used the network to group diseases based on the patterns of patient flow among them, identifying groups of closely-related diseases, even if they were not classified by the same ICD-9 chapter. Finally, we demonstrated that the network can be used to reveal trajectories of diagnoses connecting pairs of diseases that tend to co-occur in patients.

Several network-based studies of EHRs are available in the literature. However, each study evaluates a patient population that may not be representative of the general population, especially in terms of race, ethnicity, education, and income. Our study presents, to the best of our knowledge, the landscape of disease-disease relationships of the biggest cohort formed by mostly males (92%) from diverse ancestry backgrounds (e.g. Caucasians, Afro-Americans, Hispanics). However, the results obtained here will depend on the composition and domain of this specific patient cohort, suggesting that some correlations and results will not necessarily translate to the general population. Therefore, additional large-scale analysis like this on new populations have the potential to identify novel correlations that can still be highly valuable and suggest hypotheses for causality in terms of treatments, procedures, responses, and comorbidities.

Another limitation of this study comes from the potential inaccuracies in EHRs, due to systematic errors and biases in data recording. Possible errors in diagnostic codes, admission dates and incomplete recording might cause variations in the resulting disease associations^30,31^. In this study we try to overcome these limitations by filtering diagnoses with low prevalence and filtering associations that do not pass statistical significance. Because it is extremely difficult to determine when a diagnosis is a recurrence or just repeated due to the patient changing wards (or similar), here we evaluated only the first occurrence of each diagnosis. Also, the true disease state cannot be accurately assessed, which may result in biases due to systematic gaps in medical evaluation or under- and overdiagnosis. However, we highlight that our study involves a consistently larger cohort than other network-based studies in the literature, which might provide statistical power for the detection of the true signal in the data.

Comorbidities are extremely costly to individuals and health care systems and understanding the underlying determinants of comorbidity is essential to align health-care services more closely to the patients’ needs^2,3,32,33^. The approach implemented here helps us to understand the patterns of disease co-occurrence and how patients transition from one disease to another. We then go one step further to group diseases based on these same patterns, finding disease communities that could reveal mechanistic and possibly causal relationships among disorders.

We defined a framework to identify trajectories connecting any pair of diseases and represented a subset of trajectories—all trajectories connecting diabetes mellitus to cardiovascular diseases – as a network (Fig. 4). This approach allows us to have an overview of the disease trajectories, highlighting diseases present in several trajectories (high connectivity nodes) and the particular order in which diseases appear in those trajectories (link directions and network clusters). However, this representation has its own limitations, for example, not allowing the visualization of how many patients followed any particular trajectory or how relevant a given trajectory is in relation to others. Regardless, the trajectories defined in this study could help evaluate preceding diagnosis to predict the most probable next step in disease progression. They might also help on patient stratification for precision medicine and, if combined with detailed molecular-level characterization of patients, offer insights for better disease management of individuals along the course each patient may take.

Altogether, we propose a network-medicine framework that can have a direct impact on clinical practice, since it can be directly applied on EHR datasets from hospitals and healthcare providers, with the potential to offer insights to better understand high-risk diseases and progression patterns, which can help clinical resource management, policy-formulation and disease prevention.

## Methods

All methods used for this study were carried out in accordance with VA research study guidelines and regulations and by research credentialed investigators in secure, VA-approved environments. The VA Central Institutional Review Board approved these research activities acknowledging as a minimal risk data use only study, operating under HIPAA and/or informed consent waivers. All sites also approved these research activities through local Research and Development (R + D) Committees. This study is a retrospective database only study.

### Data

We retrieved all outpatient records for male patients in the Veterans Health Administration EHR database. We removed patients that were > 99 years old (as of October of 2018), resulting in over 214 million records for 9,805,451 patients. For each patient, we evaluated the first occurrence of each ICD-9 code at three-digit level. ICD-9 codes from the supplementary classification chapters (V01-V91, E000-E999) were not considered.

### Building and analyzing the disease and memory networks

We built a directed network where nodes were represented the by ICD-9 codes and the edge weights ${w}_{ij}$ represented the number of patients in which a given disease $i$ was followed by another disease $j$, with no time restriction about the time between diagnoses. To mitigate the effect of random diagnosis occurrences in a patient’s records, we used Fisher’s Exact Test to measure the statistical significance of the tendency of a disease in preceding another disease, as described in Fotouhi et al.(2018)^34^. We applied the Benjamini–Hochberg method for multiple testing correction and considered only links with adjusted p-value < 0.05. To eliminate possible errors and biases in the data, we also removed diagnosis with less than 344 patients (10th percentile of the prevalence distribution), disease pairs that occurred in less than 100 patients, and disease pairs in which one diagnosis was followed by any death diagnosis (i.e., erroneous entries in the database). For a detailed analysis of the filtered data see Supplementary Note 1. Due to the filtering steps mentioned above and that several diagnoses are gender-specific, of the 1,234 ICD-9 codes at three-digit level, 718 were included as nodes in the resulting network, which contains a total of 60,425 edges.

The relevant code for building and analyzing the TDN can be found on https://github.com/italodovalle/ehr-vha.

### Community detection

We identify network communities in the TDN by using the InfoMap^27,35^ algorithm, which identifies communities by compressing the description of how information flows in the network.

The intuition that underlies the method is that of assigning to each node in a network a code and then codifying a random walk in the network through the corresponding sequence of codes that were traversed. Real networks are characterized by communities, which the random walkers will enter and stay there for a long time, before moving to another community. This permits the use of Huffman codes to name each node in the network: there are prefix codes that are unique for each community and codes that are unique within a community but that can be reused in other communities. An analogy is the use of street names that can be reused from one city to another (e.g. each city has a Main Street, but there is no confusion because the street name is followed by the corresponding city name). The algorithm then identifies the communities by optimizing the coding of the network: too few modules will represent too many codes to represent the nodes in the network while too many communities will increase the number of prefix codes. The optional partition of the network in communities is the one that most compresses the network description. More details about the methodology can be found in Refs^27,35^.

To evaluate the profile of the communities detected by InfoMap in terms of the variety of ICD-9 chapters represented in each community, we calculated the entropy-inspired score

1 $$H=\sum \frac{-1}{{{\log}}_{2}\left(n\right)}{p}_{i} \log_{2}\left({p}_{i}\right)$$

where ${p}_{i}$ represents the proportion of diseases in the community from the chapter $i$ and $n$ represents the number of diseases in the community. The score ranges from 0, when all diseases are from the same chapter, to 1, when diseases are evenly distributed across chapters.

### Disease trajectories

To define trajectories connecting two diagnoses in the network, we first invert the edge weights (i.e., $${w}_{ij}^{-1})$$, such that lower weights indicate higher values of patients going from diagnosis *i* to *j* and result in lower distance between nodes in the network. Then, for every pair diagnosis *i* and *j*, we obtain the shortest paths connecting the pair of nodes. For example, the shortest path connecting nodes 250 and 434 is a 4-step path formed by the nodes: 250 (diabetes mellitus), 401 (essential hypertension), 436 (acute and ill-defined cerebrovascular disease), and 434 (occlusion of cerebral arteries). Finally, multiple trajectories can be aggregated by considering all disease pairs in each single trajectory (from the example above: 250–401, 401–436, 436–434) and aggregating all pairs into a network visualization (Fig. 4).

## Supplementary Information

Supplementary Information 1.

Supplementary Information 2.

## Data Availability

Final data sets underlying this study cannot be shared outside the VA, except as required under the Freedom of Information Act (FOIA) and upon request and approval through the formal mechanisms in place by the VHA Office of Research Oversight (ORO).
