# Supplementary material for: Network-medicine framework for studying disease trajectories in U.S. veterans
Source: Sci Rep. 2022 Jul 14;12:12018. doi: 10.1038/s41598-022-15764-9 (PMC9283486; doi:10.1038/s41598-022-15764-9)
Supplement: Supplementary file 1 — Supplementary Information 1. [file 41598_2022_15764_MOESM1_ESM.pdf]

Supplementary Information

Supplementary Note 1

When building the Temporal Disease Network (TDN), we removed erroneous entries, defined by disease-disease co-occurrences with less than 100 patients or co-occurrences in which the second diseases is reported after the patient is deceased. Here, we analyze the network of disease co-occurrences that were filtered out of the TDN to evaluate whether there is any specific disease category that is overrepresented among erroneous entries.

All ICD-9 codes in the dataset (n=929) are represented among the filtered co-occurrences. The codes that were filtered and that are not present in TDN (n=211) are mostly related to the following disease categories: infectious and parasitic diseases; complications of pregnancy, childbirth, and the puerperium; injury and poisoning, and conditions originating from the perinatal period (Figure S1).

We built a network only of the disease-disease co-occurrences that were filtered and compared with those in TDN. We compared the normalized weighted degree (Z-score) across disease categories, calculating a T-test p-value followed by Benjanimi-Hochberg correction between filtered and TDN networks for each disease category. We observed the following disease categories showed higher number of connections among the filtered co-occurrences (adj-pvalue < 0.05): congenital anomalies; diseases of the digestive system; diseases of the respiratory system; infectious and parasitic diseases;

injury and poisoning; neoplasms, and symptoms, signs, and ill-defined conditions (Figure S2).

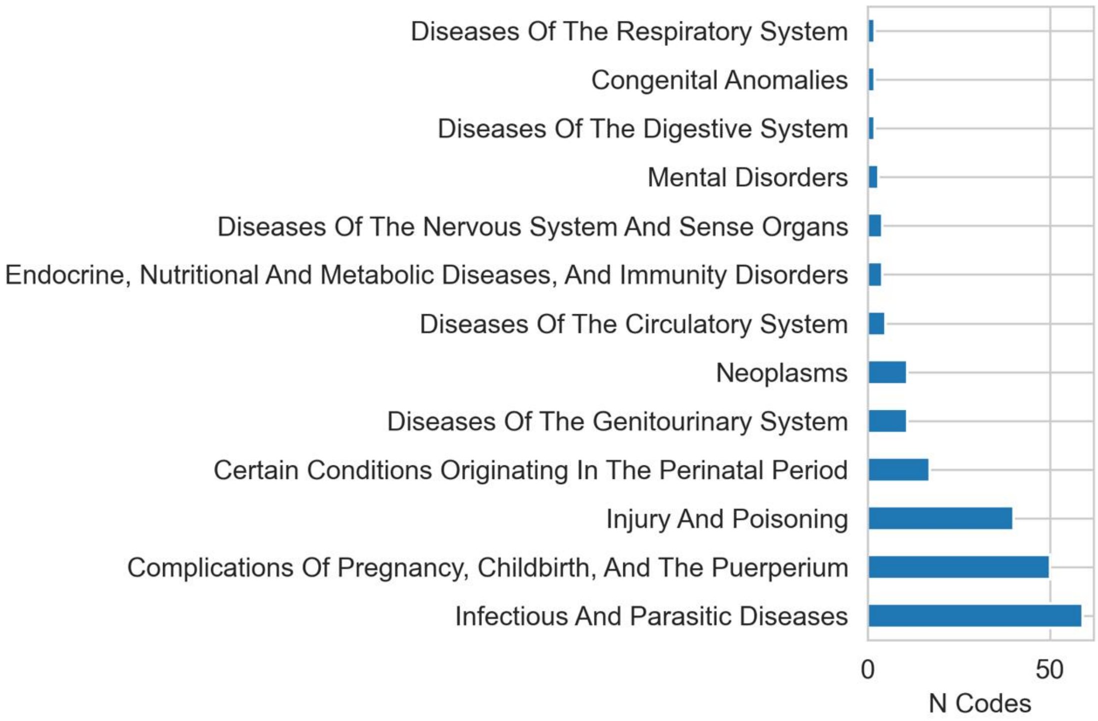

Figure S1 – Number of ICD-9 codes that were filtered out from the analysis and that are not included in the Temporal Disease Network.

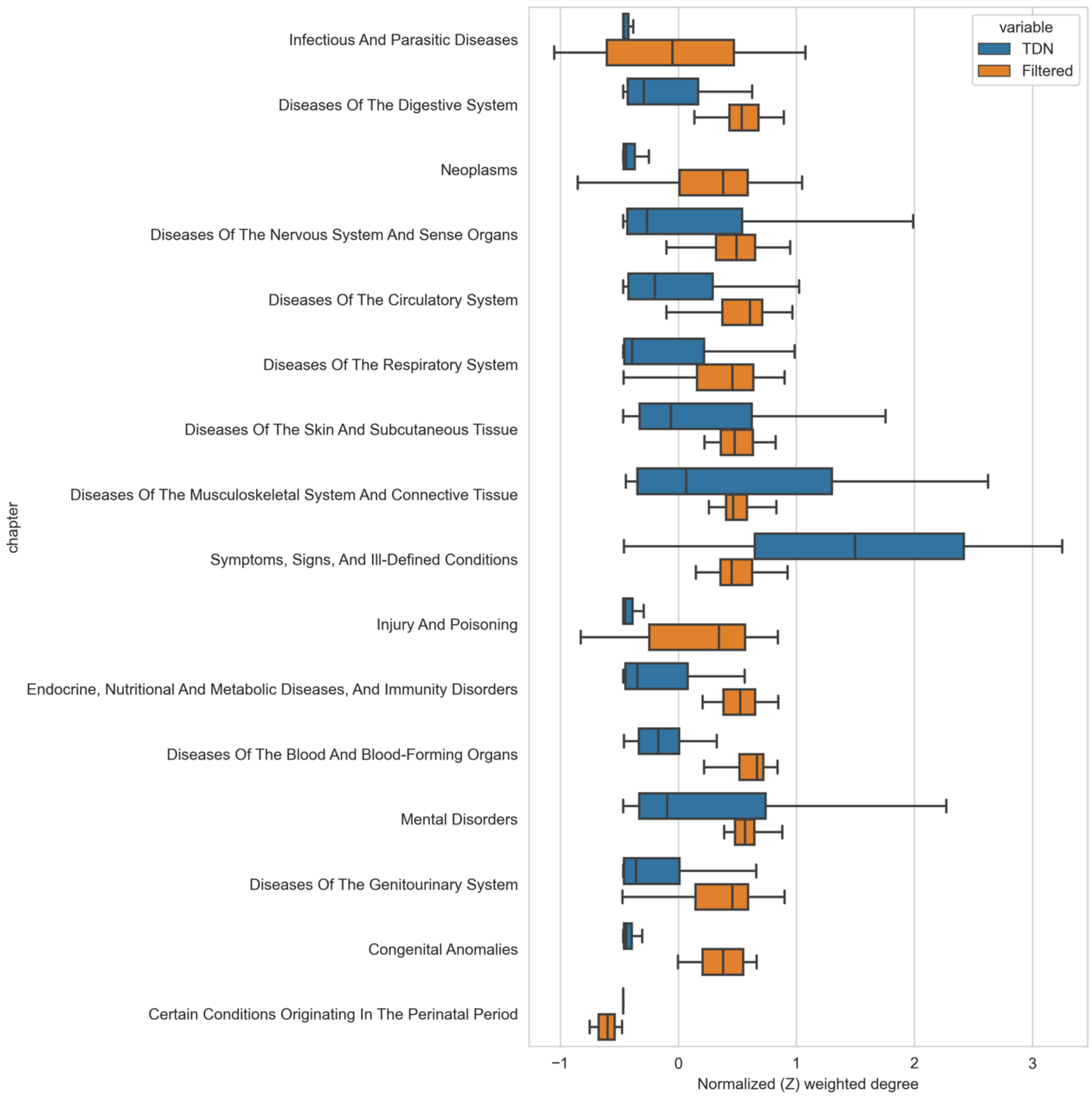

Figure S2 – Distribution of normalized weighted degree for nodes in TDN and in the network formed by disease-disease co-occurrences considered as erroneous entries in this study.

Table S1 – Comparison of previous network medicine studies evaluating disease networks based on Electronical Health Record (HER) datasets.

Reference	*Study*	*Patients*	*Nodes*	*Edges*	*Link Confidence*	*Age*	*Male/Female*	*Ethnicity*
8	Hidalgo et al 2009 A Dynamic Network Approach for the Study of Human Phenotypes	13,039,018	ICD9: 657 (3-digit) and 16,459 (5-digit)	291,172 (3-digit) and 6,088,553 (5-digit)	Relative Risk and Pearson's correlation for binary variables	65+ (Medicare)	58.3% female	90.1% white, 7.6% black, 2.3% other
9	Chmiel et al 2014 Spreading of diseases through comorbidity networks across life and gender	1,862,258	~100-500	Not reported	Chi-square test	0-8, 8-16, 16-24, 32-40, 40 - 48, 48 - 56, 56-64, 64 - 72	57.1% female	Not reported
10	Jeong et al 2017 Network-based analysis of diagnosis progression patterns using claims data	1,111,007	775	4,100	Fisher's exact followed by Bonferroni adjustment	0-85+	Not reported	Not reported
11	Westergaard et al 2019 Population-wide analysis of differences in disease progression patterns in men and women	6,909,676	1,306	27,185	Pearson correlation	All ages in national (Denmark) health registry	48.2% female	Mostly European
12	Park et al 2009 The impact of cellular networks on disease comorbidity	13,039,018		2,239	Pearson correlation with relative risk and φ-correlation	65+ (Medicare)	58.3% female	90.1% white, 7.6% black, 2.3% other
13	Klimek et al 2016 Disentangling genetic and environmental risk factors for individual diseases from multiplex comorbidity networks	1,862,258	358	Not reported	Contingency coefficient, Wilcoxon rank sum test, Benjamini-Hochberg	11 age groups 0-7, 8-15, etc	57.1% female	Not reported
14	Jensen et al 2014 Integrated Text Mining and Chemoinformatics Analysis Associates Diet to Health Benefit at Molecular Level	NA	7,178 plants, 1,613 diseases	38,090	Fisher's exact followed by Benjamini-Hochberg	NA	NA	NA
15	Beck et al 2017 Temporal order of disease pairs affects subsequent disease trajectories: The case of diabetes and sleep apnea	6,923,849	37	103	Cochran-Mantel-Haenszel test followed by Benjamini-Hochberg	Poisson regression run to predict # of comorbidities for all patients to avoid age or gender bias	Poisson regression run to predict # of comorbidities for all patients to avoid age or gender bias	Danish subjects
16	Giannoula et al 2018 Identifying temporal patterns in patient disease trajectories	643,358	Not reported	3,153 (men), 3,864 (women)	Fisher's exact followed by Bonferroni adjustment	Mean for men and women ~60	52.8% female	Regional Spanish health registry

	using dynamic time warping: A population-based study							(province of Catalonia)
17	Siggaard et al 2020 Disease trajectory browser for exploring temporal, population-wide disease progression patterns in 7.2 million Danish patients	7,186,865	928	9,608	P-values estimated using binomial distribution followed by Bonferroni adjustment	Patient matching is used to control for sex, age, discharge type, and discharge week	Patient matching is used to control for sex, age, discharge type, and discharge week. Sex-specific traits are computed using separate male/female populations	Danish subjects
18	Lee et al 2019 Inference on chains of disease progression based on disease networks	13,039,018	1,692	468,285	Ratio of relative risk	Not reported	Not reported	Not reported
19	Vlietstra et al 2020 Identifying disease trajectories with predicate information from a knowledge graph	Not reported	453	2,530	Binomial test	All ages in Danish hospital record	Not reported	Danish subjects
